# Preferential Initiation of Long-Acting Injectable Versus Oral HIV Pre-Exposure Prophylaxis Among Women Who Inject Drugs

**DOI:** 10.1093/cid/ciae450

**Published:** 2024-09-30

**Authors:** Alexis M Roth, Tyler S Bartholomew, Kathleen M Ward, Allison Groves, Silvana Mazzella, Scarlett Bellamy, K Rivet Amico, Adam W Carrico, Gail Ironson, Douglas Krakower

**Affiliations:** Department of Community Health and Prevention, Drexel University, Dornsife School of Public Health, Philadelphia, Pennsylvania, USA; Department of Public Health Sciences, University of Miami Miller School of Medicine, Miami, Florida, USA; Department of Community Health and Prevention, Drexel University, Dornsife School of Public Health, Philadelphia, Pennsylvania, USA; Department of Community Health and Prevention, Drexel University, Dornsife School of Public Health, Philadelphia, Pennsylvania, USA; Prevention Point Philadelphia, Philadelphia, Pennsylvania, USA; Department of Biostatistics, Boston University School of Public Health, Boston, Massachusetts, USA; Department of Health Behavior and Health Education, University of Michigan School of Public Health, Ann Arbor, Michigan, USA; Department of Health Promotion and Disease Prevention, Florida International University, Robert Stempel College of Public Health and Social Work, Miami, Florida, USA; Department of Psychology, University of Miami, Miami, Florida, USA; Department of Population Medicine, Harvard Medical School, Boston, Massachusetts, USA; Division of Infectious Diseases, Beth Israel Deaconess Medical Center, Boston, Massachusetts, USA

**Keywords:** HIV prevention, pre-exposure prophylaxis, women who inject drugs, long-acting injectable cabotegravir

## Abstract

Fifty-five of 62 women who inject drugs (WWID) selected long-acting cabotegravir (CAB-LA) over oral PrEP, and 51/55 received a first injection. More recent injection drug use and number of sexual partners were associated with selecting CAB-LA (*P* < .05). Findings provide preliminary evidence of a strong preference for longer-acting products among WWID.

Injection drug use has increased dramatically in the United States; the estimated number of adults injecting drugs increased from 775 000 in 2011 to more than 3 600 000 in 2018 [1, 2]. This surge has coincided with the first increase in human immunodeficiency virus (HIV) incidence attributable to injection drug use in 2 decades [3]. Women who inject drugs (WWID) have been disproportionately represented among these new cases [4], suggesting they would benefit from increased access to HIV pre-exposure prophylaxis (PrEP). Yet, PrEP remains under-prescribed to WWID, representing only 0.15% of new prescriptions [5]—in part, due to providers' perceptions that people who inject drugs may have lower adherence to oral PrEP and thus derive less protection [6]. Long-acting injectable cabotegravir (CAB-LA) received regulatory approval for use as PrEP in the United States in 2021 and offers an effective alternative to oral PrEP [7]. Because CAB-LA is administered monthly for 2 months then bimonthly, this modality may provide a more effective option for individuals who have difficulty with pill-taking, similar to the success with other long-acting injectables (eg, injectable buprenorphine for opioid use disorder, medroxyprogesterone acetate as contraception [8]). While research has shown that WWID find long-acting injectable PrEP acceptable [9], data regarding its use remain sparse. To address this gap, we analyzed PrEP prescription data from a subsample of WWID participating in an ongoing randomized controlled trial (called Efficacy of a Trauma Intervention for Affect Regulation, Adherence, and Substance Use to Optimize PrEP for Women Who Inject Drugs [TIARAS]) designed to reduce HIV acquisition risk [10].

## METHODS

The TIARAS study enrolled English-speaking, cis-gender women who were at least 18 years of age, injected drugs within the past 6 months, and had received a PrEP prescription within the past month at Prevention Point Philadelphia (PPP), the largest syringe services program in the mid-Atlantic region of the United States. PrEP prescriptions were obtained prior to enrollment in TIARAS. The process begins with an individualized PrEP counseling session with PPP navigators, formulation selection (CAB-LA or daily oral), and relevant PrEP clinical screening (HIV RNA, serum creatinine, and sexually transmitted infections). Per PPP's standard of care, women selecting oral PrEP met with a prescriber for clinical evaluation and were dispensed medications. Those selecting CAB-LA returned approximately 7 days later for a clinical evaluation and their first injection. Medication costs were paid by each participant's insurance, and those without health insurance worked with PPP case managers to obtain coverage.

### Variables and Measures

Data sources included participant medical records and a detailed self-administered survey. Data on selected modality for PrEP were extracted from electronic medical records at PPP and could be either oral PrEP or CAB-LA. All participants selecting oral PrEP were observed taking their first dose. All participants selecting injectable CAB-LA were scheduled to receive their first and subsequent injections at PPP. This allowed for extraction of PrEP initiation data from the medical records for both oral and injectable PrEP, defined specifically as having been observed taking the first oral PrEP dose or having received the first CAB-LA injection.

From the TIARAS baseline survey, sociodemographic information, drug use and sexual behaviors, mental and physical health functioning, and healthcare utilization were characterized. For these descriptions and subsequent analyses, we recategorized sociodemographic factors, including sexual orientation (recategorized as heterosexual vs homosexual/bisexual), frequency of skipping meals in the past 3 months due to finances (almost weekly vs less than weekly), and current living situation (stably housed [apartment/house owned/rented by participant, partner, family, or friend] vs unhoused [shelter, car, street, abandoned building, or facility]). Annual income (continuous) was a composite of various income sources: full-/part-time work, social security payments, public assistance, and odd jobs/hustling (eg, selling sex and shoplifting).

Other measures assessed patterns of alcohol/drug consumption in the past 3 months [11]. We created a polydrug use variable that included opioid use only (street opioids [fentanyl, heroin], or nonmedical use prescription opioids), stimulant use only (cocaine, methamphetamines, or prescription), or both. We also assessed the frequency of injection drug use in the past month (daily, weekly, monthly, or none), average daily number of injections, receptive sharing (yes vs no) of syringes and paraphernalia (drug, cookers, cotton, rinse water, piggybacking) within the last month, and lifetime overdose history (ever vs never). Sexual behavior over the past 6 months included the following: number of sexual partners, condom use during vaginal or anal sex within the past month (inconsistent [never, rarely, sometimes, most of the time] vs consistent [always]), sex exchange (trading or selling oral, vaginal or anal sex for money, food, drugs or favors; yes vs no), and bacterial sexually transmitted infections (STIs) (syphilis, gonorrhea, chlamydia) at the most recent visit (yes vs no). Depression and anxiety were measured within the last 7 days [12]. Scores were summed (range, 5–20) and classified within normal limits (<8), mild (8–11, depression; 8–10, anxiety), moderate (12–16, depression; 11–15, anxiety), or severe (17–20, depression; 16–20, anxiety) [12]. Healthcare engagement (yes vs no) included emergency department use, hospital admissions, medical provider office visits, and taking medication to treat opioid use disorder in the past 6 months. Finally, any prior PrEP use (yes vs no) and modality (oral, injectable, or both) were assessed.

### Analyses

Participant responses were characterized with frequencies and corresponding percentages for categorical variables and medians and interquartile ranges (IQRs) for continuous variables. Potential differences in sociodemographic variables between participants who selected oral PrEP and those who selected CAB-LA were assessed using Fisher's exact test for categorical variables and Wilcoxon exact rank sum test for continuous variables. All analyses were performed using SAS version 9.4. statistical software (SAS Institute, Cary, NC, USA), and significance level was set at a 2-tailed alpha of .05.

### Ethical Considerations

All participants provided written informed consent and received $30 US dollars as compensation for their baseline visit. In addition, as part of an incentive program to encourage engagement in their PrEP program, participants received $50 from PPP prior to enrolling in the trial. The Drexel University and City of Philadelphia Department of Public Health institutional review boards and the PPP research review board approved the study procedures.

## RESULTS

The sample (N = 62) includes primarily middle-aged (median, 42 years), non-Hispanic White (68%) women. As presented in Table 1, most women were homeless (69%) and engaged in polysubstance use (97%), with a high frequency of injection drug use, such that 82% of women reported daily use and injecting approximately 6 times per day. Syringe sharing (33%) and sex exchange (48%) were common, and approximately 1 in 10 women reported a bacterial STI diagnosis within the past 6 months. Most women (77%) had engaged in some type of medical care in the past 6 months (emergency department, primary care provider, or hospital admission). Just over one-third of WWID (37%) were prescribed PrEP prior to this study. Among women with PrEP experience (n = 19), 6 had taken oral PrEP, 7 had received CAB-LA, and 6 had experience with both.

**Table 1.** Demographic and Behavioral Factors Associated With PrEP Modality Selection Among Women Who Inject Drugs Accessing Care Within a Syringe Services Program

	Total	Oral PrEP	Injectable PrEP
PrEP modality selected	62	7 (11%)	55 (89%)
Sociodemographic characteristics			
Median age (IQR)	42 (36, 47)	47 (42, 49)	41 (35, 46)
Race (n = 60)			
American Indian/Alaska Native	2	…	2 (100%)
Black or African American	11	2 (18.2%)	9 (81.8%)
White	42	4 (9.5%)	38 (90.5%)
More than 1 race	5	1 (20.0%)	4 (80.0%)
Ethnicity			
Hispanic	7	…	7 (100%)
Non-Hispanic	57	7 (12.7%)	48 (87.3%)
Sexual orientation			
Heterosexual/straight	46	7 (15.2%)	39 (84.8%)
Homosexual/lesbian	3	…	3 (100%)
Bisexual	13	…	13 (100%)
Currently in partnered relationship	22	1 (4.5%)	21 (95.5%)
Educational attainment			
Less than high school	12	3 (25%)	9 (75%)
High school graduate/GED	21	1 (4.8%)	20 (95.2%)
Some college or higher	29	3 (10.3%)	26 (89.7%)
Median annual income (IQR)	$8700 ($3840–$17 376)	$7992 ($2400–$17 892)	$9000 ($3840–$17 376)
Skipped meal due to finances 3 mo			
Almost weekly	42	6 (13.9%)	37 (86.1%)
Less than weekly	19	1 (5.3%)	18 (94.7%)
Current living situation			
Apartment/house owned/rented by participant, partner, family, or friends	19	3 (15.8%)	16 (84.2%)
Homeless shelter	13	2 (15.4%)	11 (84.6%)
Car, street, or abandoned building	28	2 (7.1%)	26 (92.9%)
Other (facility)	2	…	2 (100%)
Substance use and related outcomes			
Polysubstance use within 3 mo (n = 60)			
Stimulants only	…	…	…
Opioids only	2	1 (50%)	1 (50%)
Both stimulants and opioids	58	6 (10.3%)	52 (89.7%)
Frequency of injection drug use within month (n = 61)^a^			
None	4	…	4 (100%)
Weekly or monthly	7	3 (42.9%)	4 (57.1%)
Daily	50	4 (8.0%)	46 (92.0%)
Average daily injections (n = 50)	6.5 (4, 10)	9 (6, 10)	6 (4, 8)
Receptive syringe sharing within month			
Yes	20	2 (10.0%)	18 (90.0%)
No	42	5 (12.0%)	37 (88.0%)
Paraphernalia sharing within month^b^			
Yes	47	3 (6.5%)	43 (93.5%)
No	16	4 (25.0%)	12 (75.0%)
Lifetime overdose history			
Ever	51	6 (11.8%)	45 (88.2%)
Never	11	1 (9.1%)	10 (90.9%)
Sexual behavior and related outcomes			
Median sex partners within month (IQR)^a^	1 (0, 6)	0 (0, 1)	2 (0, 10)
Condom use within month			
Inconsistent	21	…	21 (100%)
Consistent	41	7 (17.1%)	34 (82.9%)
Sex exchange within 6 mo (n = 61)			
Yes	29	1 (3.5%)	28 (96.5%)
No	32	6 (18.7%)	26 (81.3%)
Bacterial STI within 6 mo			
Yes	8	…	8 (100%)
No	55	7 (13.0%)	47 (87.0%)
Health status and healthcare utilization			
Prior PrEP use (n = 60)			
PrEP experienced	22	1 (4.5%)	21 (95.5%)
PrEP naïve	38	5 (13.2%)	33 (86.8%)
Prior PrEP modalities used (n = 19^c^)			
Oral pill	6	…	6 (100%)
CAB-LA	7	…	7 (100%)
Both	6	1 (16.7%)	5 (83.3%)
Healthcare utilization within 6 mo	29	4 (13.8%)	25 (86.2%)
Medication for opioid use disorder within 6 mo (n = 61)	22	2 (9.1%)	20 (90.9%)
Emergency department visit within 6 mo	31	4 (12.9%)	27 (87.1%)
Hospitalized within 6 mo^b^	23	5 (21.7%)	18 (78.3%)
Depression (past week)			
Within normal limits	6	…	6 (100%)
Mild	10	…	10 (100%)
Moderate	32	3 (9.4%)	29 (90.6%)
Severe	14	4 (28.6%)	10 (71.4%)
Anxiety (past week)			
Within normal limits	4	…	4 (100%)
Mild	10	…	10 (100%)
Moderate	27	4 (14.8%)	23 (85.2%)
Severe	21	3 (14.3%)	18 (85.7%)

The table displays row percentages. N = 62.

Abbreviations: CAB-LA, long-acting cabotegravir; GED, General Educational Development; IQR, interquartile range; PrEP, pre-exposure prophylaxis; STI, sexually transmitted infection.

^a^ *P* ≤ .05.

^b^ *P* ≤ .10.

^c^Three participants elected not to indicate PrEP formulation.

### PrEP Choice

Most participants selected CAB-LA over oral PrEP (89%; n = 55). While no demographic factors were significantly associated with PrEP modality selection, HIV risk behaviors were. Women selecting CAB-LA reported an average of 2 sexual partners within 6 months compared to none among those selecting oral PrEP (*P = .*01). Injection drug frequency was also associated with selecting CAB-LA (*P = .*05). For example, 92% of women reporting daily injection drug use chose CAB-LA, as did the majority of women reporting past week or past month injection drug use (57%).

### PrEP Initiation

All women in the group selecting oral PrEP had documentation of directly observed ingestion of the first oral dose (7/7), and most received a first CAB-LA loading dose (58/62; 94%).

## DISCUSSION

This study describes product choice among a sample of WWID enrolled in PrEP care within a community-based syringe services program. Most selected CAB-LA and returned for their first dose. Additional studies should assess whether delayed initial injection visits among WWID correlate with future missed injections with CAB-LA, given the potential to select for integrase-strand transfer inhibitor resistance for individuals who acquire HIV with subtherapeutic cabotegravir levels; this scenario may be of particular concern for women, because the pharmacokinetic tail for CAB-LA may be longer in women than in men [13].

Notable strengths of this study are its focus on WWID, who are notably underrepresented in PrEP research, and on PrEP initiation since most studies have been based on hypothetical choices, willingness, or intentions. By design, our paper focuses on product choice and initiation among women already engaging in care. Thus, we are unable to describe why women choose to initiate PrEP, how they selected a particular product, or adherence. This research is much needed as are interventions to increase PrEP prescribing to this group, given that most women were PrEP naïve despite having an indication for PrEP, and had reported recent healthcare utilization. Overall, our findings provide preliminary evidence that WWID prefer CAB-LA, highlight the importance of expanding PrEP offerings as a critical component of patient-centered PrEP care, and are a reminder that clinicians need to personalize their PrEP recommendations based on women's patterns of engagement in care to optimize benefits while minimizing the risk of drug resistance.

## Notes

***Author Contributions.*** Conceptualization and methodology (A. M. R., T. S. B., A. G., S. B., K. R. A., A. W. C., G. I., D. K.); formal analysis and data curation (T. S. B., K. M. W., S. B.); writing—original draft (A. M. R.); writing—review and editing (T. S. B., K. M. W., A. G., S. M., S. B., K. R. A., A. W. C., G. I. , D. K.); supervision and funding acquisition (A. M. R.).

***Acknowledgments.*** Research staff on TIARAS: Kerry Miley, Kathleen Ward, Erin McDowell, Tamica Matos, Katie Singley, Sneha Kamarajugadda, Imani Ali, Julianna Berardi, Sidney Palmer, Ally D’Angelo, Fahamida Khanam, Elana Forman, Andrea McCord, Danielle Whyte, Gabriella Ryan. PrEP staff at PPP: Kareem Mims, Desmond Doyley, Alfonso Melendez, Joy Parker, Rafael Jimenez, Silvana Mazella, Andres Friere, Anne Kelly, Jessica Misner, Daniel Smith, Kristi Petrillo, and José Benitez.

***Financial support*.** This work was supported by National Institutes of Health (grant number R01DA054543; to A. M. R.) and ViiV Healthcare (A. M. R.).
